# Plasma extracellular vesicle long RNA profiles in the diagnosis and prediction of treatment response for breast cancer

**DOI:** 10.1038/s41523-021-00356-z

**Published:** 2021-12-10

**Authors:** Yonghui Su, Yuchen Li, Rong Guo, Jingjing Zhao, Weiru Chi, Hongyan Lai, Jia Wang, Zhen Wang, Lun Li, Yuting Sang, Jianjing Hou, Jingyan Xue, Zhimin Shao, Yayun Chi, Shenglin Huang, Jiong Wu

**Affiliations:** 1 Department of Breast Surgery, Fudan University Shanghai Cancer Center, and the Shanghai Key Laboratory of Medical Epigenetics, Institutes of Biomedical Sciences, Fudan University, Shanghai, 200032 P. R. China; 2 Shanghai Key Laboratory of Breast Cancer, Fudan University Shanghai Cancer Center, Fudan University, Shanghai, 200032 P. R. China; 3 Department of Oncology, Shanghai Medical college, Fudan University, Shanghai, 200032 P. R. China; 4 Shanghai Key Laboratory of Radiation Oncology, Fudan University Shanghai Cancer Center, Shanghai, 200032 P. R. China

**Keywords:** Diagnostic markers, Breast cancer

## Abstract

A large number RNAs are enriched and stable in extracellular vesicles (EVs), and they can reflect their tissue origins and are suitable as liquid biopsy markers for cancer diagnosis and treatment efficacy prediction. In this study, we used extracellular vesicle long RNA (exLR) sequencing to characterize the plasma-derived exLRs from 112 breast cancer patients, 19 benign patients and 41 healthy participants. The different exLRs profiling was found between the breast cancer and non-cancer groups. Thus, we constructed a breast cancer diagnostic signature which showed high accuracy with an area under the curve (AUC) of 0.960 in the training cohort and 0.900 in the validation cohort. The signature was able to identify early stage BC (I/II) with an AUC of 0.940. Integrating the signature with breast imaging could increase the diagnosis accuracy for breast cancer patients. Moreover, we enrolled 58 patients who received neoadjuvant treatment and identified an exLR (exMSMO1), which could distinguish pathological complete response (pCR) patients from non-pCR with an AUC of 0.790. Silencing MSMO1 could significantly enhance the sensitivity of MDA-MB-231 cells to paclitaxel and doxorubicin through modulating mTORC1 signaling pathway. This study demonstrated the value of exLR profiling to provide potential biomarkers for early detection and treatment efficacy prediction of breast cancer.

## Introduction

Breast cancer (BC) is the most common malignancy in women worldwide^1^. As with many cancers, BC found at an early stage carries much-improved prognosis compared to advanced stage disease^2^. Thus, the detection and efficient treatment of early stage BC has significant potential for reducing its mortality. Currently, mammography and ultrasound are the optimal methods for BC screening and are recommended by different clinical guidelines. Unfortunately, their sensitivity and specificity are not consistent among different studies^3–7^. Carbohydrate antigen 15-3 (CA15-3) and carcino-embryonic antigen (CEA) are blood-based biomarkers that are currently used for BC screening and treatment response monitoring; however, their sensitivity and specificity remain poor^8^. Hence, new efficient diagnostic methods and treatment efficacy predictive approaches for early stage BC must be developed.

Because of the existence of tumors, pre-surgery (neoadjuvant) chemotherapy (NACT) was used in many clinical trials to evaluate the treatment response. The latest blood-based biomarkers, such as circulating tumor cells (CTCs), circulating tumor DNA (ctDNA), and tumor-derived extracellular vehicles (EVs), have the potential of assessing real-time tumor responses to therapy as well as identifying dynamically resistant clones in BC which undergo NACT^9–12^. A meta-analysis published by Bidard et al.^13^ suggested that the CTC count is an independent and quantitative prognostic factor for early BC patients treated by NACT. Magbanua et al.^11^ found that the ctDNA clearance during NACT of high-risk early BC was a significant predictor of response and metastatic recurrence. EVs, including exosomes and microvesicles, are lipid bilayer-enclosed structures that contain various cargoes, including large number RNA species^14,15^. Rodríguez-Martínez et al.^16^ demonstrated that the exosomal microRNAs (miRNAs) profile could act as a predictive tool in localized BC undergo NACT. Besides miRNAs, certain long RNAs are enriched and stable in EVs, and they could reflect their tissue origins and are potential suitable as liquid biopsy markers for cancer diagnosis and treatment efficacy monitoring^17,18^. However, the roles of EV long RNAs (exLRs) profile in BC, especially in BC diagnosis and treatment efficacy prediction, remain unknown.

In this study, we performed exLR-seq on plasma samples collected from 172 subjects, including BC patients, breast benign disease patients and healthy individuals who were receiving routine healthcare with the aim of exploring the potential of exLR-based signature as a clinically actionable biomarker for BC diagnosis. Moreover, we evaluated the treatment monitoring role of exLRs profile in BC patients who were receiving NACT.

## Results

### Patient characteristics

One hundred and seventy-two individuals were included in this study; the participants consisted of 112 BC patients, 19 benign patients, and 41 healthy donors (Table 1). Among the 112 BC patients, 28 were at stage I, 35 were at stage II, 15 were at stage III, and 34 were at stage IV (Table 1). Forty-nine BC patients with stage II or III received neoadjuvant chemotherapy in our cohort. The benign patients included 10 adenosis, 4 fibroadenomas, 4 mastitis, and 1 intraductal papilloma. Other clinical features, including the age, the Breast Imaging Reporting and Data System classification (BI-RADS) of ultrasound or mammography, plasma CA15-3 and CEA level, are shown in Table 1.

**Table 1:** Patient characteristics.

	Breast cancer	Breast benign disease	Healthy cohort
Total	112	19	41
Age, years			
Median	51.5	46	54
Range	31–75	22–61	40–85
Mammography (BI-RADS)*			
1–3	19 (20.7%)	3 (21.4%)	/
4	47 (55.2%)	11 (78.6%)	/
5	20 (24.1%)	0 (0%)	/
Ultrasound (BI-RADS)*			
1–3	1 (1%)	7 (43.8%)	/
4	50 (50.5%)	9 (56.2%)	/
5	48 (49.5%)	0 (0%)	/
Serum CA15-3, U/ml*			
≤25	58 (70.7%)	/	/
>25	24 (29.3%)	/	/
Serum CEA, ng/ml*			
≤5.2	59 (72.0%)	/	/
>5.2	23 (28.0%)	/	/
Cancer stage			
I	28 (25.0%)	/	/
II	35 (31.3%)	/	/
III	15 (13.4%)	/	/
IV	34 (30.4%)	/	/
Hormone receptor			
Positive	63 (56.3%)	/	/
Negative	49 (43.8%)	/	/
HER2*			
Positive	64 (57.7%)	/	/
Negative	47 (42.3%)	/	/

Abbreviations: *BI-RADS* breast imaging reporting and data system, *CA15-3* carbohydrate antigen 15-3, *CEA* carcino-embryonic antigen, *HER2* human epidermal growth factor receptor 2.

*Excluded the unknown category.

### EV isolation and exLR-seq

**Fig. 1:**
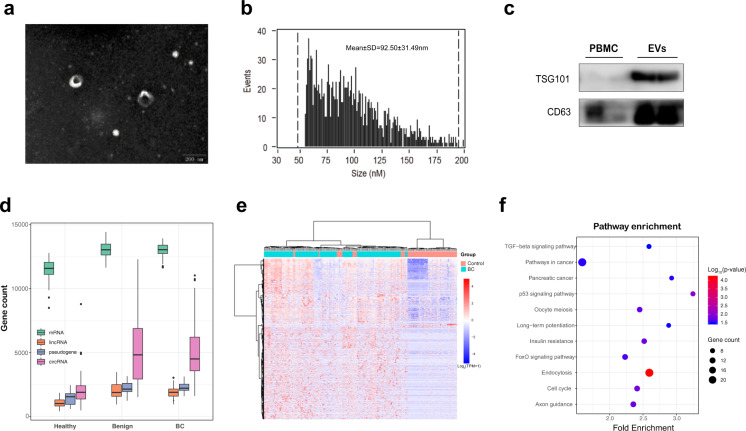
ExLR profiles of the cohort. **a**, **b** EVs were detected by transmission electron microscopy (**a**) and flow cytometry (**b**). Scale bar, 200 nm. **c** Western blots of EV markers TSG101 and CD63 expression in peripheral blood mononuclear cells (PBMC) and isolated vesicles. **d** The distribution of exLRs per sample among BC, benign, and healthy patients. **e** Heatmap of unsupervised hierarchical clustering of the exLRs that were differentially expressed between BC patients and controls (healthy + benign). Each column represents an individual sample, and each row represents an exLR. The scale represents the expression values. **f** KEGG pathway enrichment analysis for the differentially expressed exLRs.

The isolated vesicles were rounded, cup-shaped, and membrane-enclosed, as observed by transmission electron microscopy (TEM, Fig. 1a). Flow cytometry revealed a heterogeneous population of spherical nanoparticles, with abundant peaks of less than 200 nm and a mean diameter of 92.5 nm (Fig. 1b). Western blot analysis revealed that the exosomal characteristic markers CD63 and TSG101 were enriched in the isolated vesicles but not in peripheral blood mononuclear cells (PBMCs, Fig. 1c). These findings indicate that the isolated EVs consisted mostly of exosomes.

ExLR-seq was conducted using plasma samples from 172 healthy individuals and patients. Approximately 15,000 annotated genes, including mRNAs, lncRNAs pseudogenes, and circRNAs, were reliably detected in each sample. More mRNAs, lncRNAs, pseudogenes, and circRNAs were identified in benign and BC groups than healthy group (Fig. 1d). We identified 1552 exLRs that were differentially expressed in BC samples compared with controls (benign + healthy) by the Mann–Whitney U test (false discovery rate (FDR) < 0.01, |fold change (FC) | > 1.5). Most different exLRs were up-regulated in the BC group. Unsupervised hierarchical clustering revealed a clear separation of the BC and control group (Fig. 1e). The Kyoto Encyclopedia of Genes and Genomes (KEGG) pathway analysis revealed that differentially expressed exLRs were enriched for some pathways involved in cancer, such as the TGF-beta signaling pathway, pathways in cancer, pancreatic cancer, and p53 signaling pathway (Fig. 1f).

### Blood exLRs may reflect the relative fractions of different cell types

**Fig. 2:**
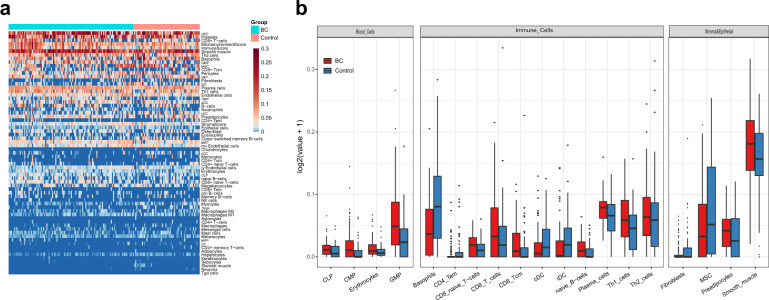
Relative fractions of different cell types by exLR-seq in BC. **a** ExLRs reflect the relative proportions of different cell types using xCell. Each column represents an individual sample, and each row represents a cell type. The scale represents the relative fractions. **b** Comparison of different cell types from the exLR-seq data between BC and controls. Only significant differences between these two groups are shown.

Since blood EVs are derived from a variety of tissues, we further used the xCell software (http://xcell.ucsf.edu) to characterize source contribution of the cell fractions from the exLR-seq profiles. xCell is a web tool that performs cell type enrichment analysis from gene expression data for 64 immune and stroma cell types. The relative proportion of the different cell types in BC and control samples are shown in Fig. 2a. We then adjusted the cell type enrichment scores to cell type proportions; 19 normalized average xCell scores were significantly different between BC and control groups (Fig. 2b). Blood cells (common lympoid progenitors (CLP), common myeloid progenitor cell (CMP), erythrocytes and granulocyte macrophage progenitor (GMP)), immune cells (CD8 + T-cells, naïve B-cells, plasma cells, Th1 and Th2 cells), and stroma/epithelial cells (preadipocytes and smooth muscle cells) were significantly enriched in BC group compared with control (Fig. 2b).

### Establishment of an exLR d-signature for BC

**Fig. 3:**
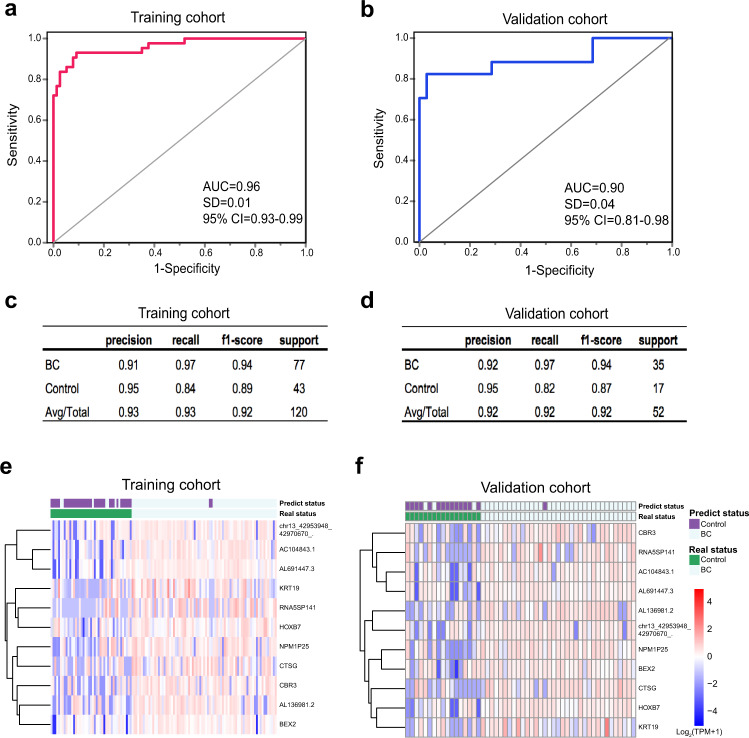
Blood exLR profiles can distinguish BC patients from controls. **a**, **b** ROC for the performance of the exLR d-signature in the training (*n* = 120, **a**) and validation (*n* = 52, **b**) cohorts. **c**, **d** The diagnosis effects of the d-signature in the training (**c**) and validation (**d**) cohorts. **e**, **f** Unsupervised hierarchical clustering of eleven exLRs selected for use in the d-signature in the training (**e**) and validation (**f**) cohorts. Each column represents an individual sample, and each row represents an exLR. The scale represents the expression values. ROC receiver operating characteristic, AUC area under the curve, SD standard deviation, CI confidence interval.

The different exLRs profiling between the BC and control groups implies that the exLRs have potential as biomarkers for the detection of BC. We then explored an exLR d-signature for the diagnosis of BC. ExLRs (*n* = 1511) that were upregulated in BC patients compared with controls were selected using a training cohort of 43 control individuals and 77 BC patients. The selected exLR markers were analyzed using the random forest algorithm and the LASSO method to shrink the number of variables. Finally, eleven exLR markers (BEX2, AC104843.1, AL136981.2, KRT19, NPM1P25, CTSG, CBR3, HOXB7, AL691447.3, RNA5SP141, and circRNA chr13_42953948_42970670_-) were selected and used to construct a BC classifier (Supplementary Table 1). Using the support vector machine (SVM) algorithm, we established a diagnostic model and generated an exLR d-signature for BC. The exLR d-signature comprising the eleven exLRs distinguished the BC patients from controls with an areas under the curve (AUC) of 0.96 (95% confidence interval (CI): 0.93 to 0.99, standard deviation (SD): 0.01), a precision of 0.93, a recall of 0.93 and a f1-score of 0.92 in the training cohort (Fig. 3a, c). The diagnostic accuracy was 92.5%. The exLR d-signature was then applied to the validation cohort; BC was detected with an AUC of 0.90 (95% CI: 0.81 to 0.98. SD: 0.04), a precision of 0.92, a recall of 0.92 and a f1-score of 0.92 (Fig. 3b, d). The diagnostic accuracy was 92.3%. Unsupervised hierarchical clustering using the eleven exLRs effectively distinguished BC from controls with high specificity and sensitivity (Fig. 3e, f).

### The exLR d-signature detects early BC

**Fig. 4:**
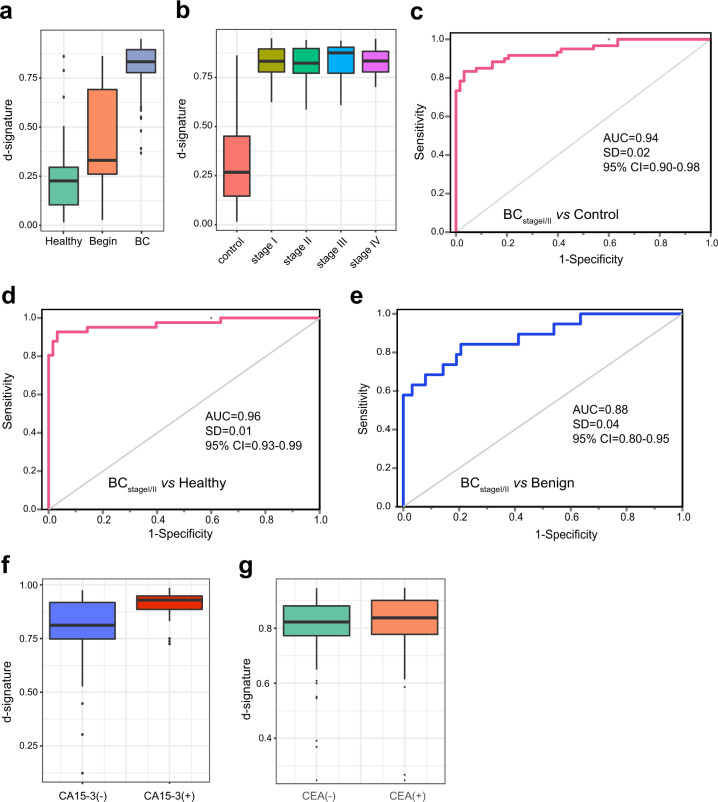
ExLR-based d-signature for the diagnosis of early stage BC. **a** ExLR d-signature scores in healthy (*n* = 41), benign (*n* = 19), and BC (*n* = 112). **b** ExLR d-signature scores in BC patients with stage I (*n* = 28), II (*n* = 35), III (*n* = 15), and IV (*n* = 34). **c**–**e** ROC for the performance of the exLR d-signature in BC with early stages (Stage I/II) compared to control (**c**), healthy (**d**), and benign (**e**). **f**, **g** ExLR d-signature scores in BC patients with different serum CA15-3 (**f**) and CEA (**g**) statuses. AUC area under the curve, SD standard deviation, CI confidence interval.

Early stage BC diagnosis allows immediate surgery without prior chemotherapy or radiation therapy and has a favorable prognosis. We found that BC exhibited a high median exLR d-signature score when compared with benign disease in the entire cohort (0.809 vs 0.444; Mann–Whitney U test, *p* < 0.001) and healthy (0.809 vs 0.254; Mann–Whitney U test, *p* < 0.001, Fig. 4a). We also observed no correlation between the d-signature scores and tumor stages (Fig. 4b), which suggests that the diagnostic performance of the d-signature was independent of the tumor burden, which would make it an optimal diagnostic tool for the detection of BC. Therefore, we next confirmed the diagnostic performance of the d-signature in early stage of BC. The d-signature can identify early stage (I/II) BC from controls with an AUC of 0.94 (95% CI: 0.90 to 0.98), a precision of 0.89, a recall of 0.89, and a f1-score of 0.88 (Fig. 4c). Furthermore, the d-signature can identify early stage BC from healthy and benign groups with an AUC of 0.96 (95% CI: 0.93 to 0.99, Fig. 4d) and 0.88 (95% CI: 0.80 to 0.95, Fig. 4e), respectively. These results demonstrated that the exLR d-signature could be used for high-accuracy diagnosis of BC, even for early BC.

CA15-3 and CEA are the biomarkers that are currently used for BC screening and recurrence monitoring. The d-signature scores were significantly higher in the BC patients with CA15-3 positive compared to negative patients (Fig. 4f). However, there is no correlation between the d-signature score and CEA status (Fig. 4g).

### The exLR d-signature has improved diagnostic performance for BC detection

**Fig. 5:**
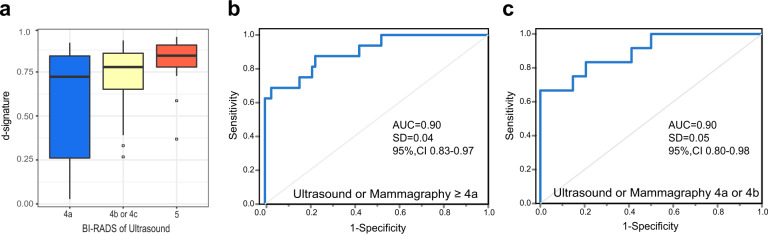
Combined imaging results with exLR-based d-signature for BC diagnosis. **a** ExLR d-signature scores in BC patients with different BI-RADS scores. **b**, **c** ROC for the performance of exLR d-signatures in BC with ultrasound or mammography ≥ 4a (**b**) and 4a or 4b (**c**).

The ability to complement the limitations of the current imaging examination in the detection of BC would add value to a biomarker for the diagnosis of BC. We found that BC with imaging BI-RADS 5 exhibited a higher median exLR d-signature score when compared with BI-RADS 4b/4c and BI-RADS 4a (Fig. 5a). Clinically, in general, the probability of a malignant tumor for patients with imaging BI-RADS 4 is estimated to be within a range of 2–95%. In our cohort, 101 and 46 patients were with imaging BI-RADS ≥ 4a (including 4a, 4b, 4c, and 5) and BI-RADS 4a/4b, 85 and 46 patients were diagnosis with BC, with diagnosis accuracy of 81.6% and 73.9%, respectively. We combined the exLR d-signature with the corresponding BI-RADS scores for predicting the presence of cancer. The diagnosis accuracy was approximately 91.9%, with a precision of 0.92, recall of 0.92, f1-score of 0.92, and AUC of 0.90 (95% CI: 0.83 to 0.97, SD: 0.04) (Fig. 5b) for patients with BI-RADS ≥ 4a. If only the patients with BI-RADS 4a or 4b were considered, the integrated predictive value for predicting the presence of cancer in these patients was approximately 91.3%, with a precision of 0.92, recall of 0.91, f1-score of 0.91, and AUC of 0.90 (95% CI: 0.80 to 0.98, SD: 0.05) (Fig. 5c). These results indicate that exLRs combined with the BI-RADS system could be utilized as a more accurate biomarker for differential diagnosis of early-stage BC compared with the BI-RADS system only.

### Plasma exMSMO1 as predictive biomarker for neoadjuvant chemotherapy of BC

**Fig. 6:**
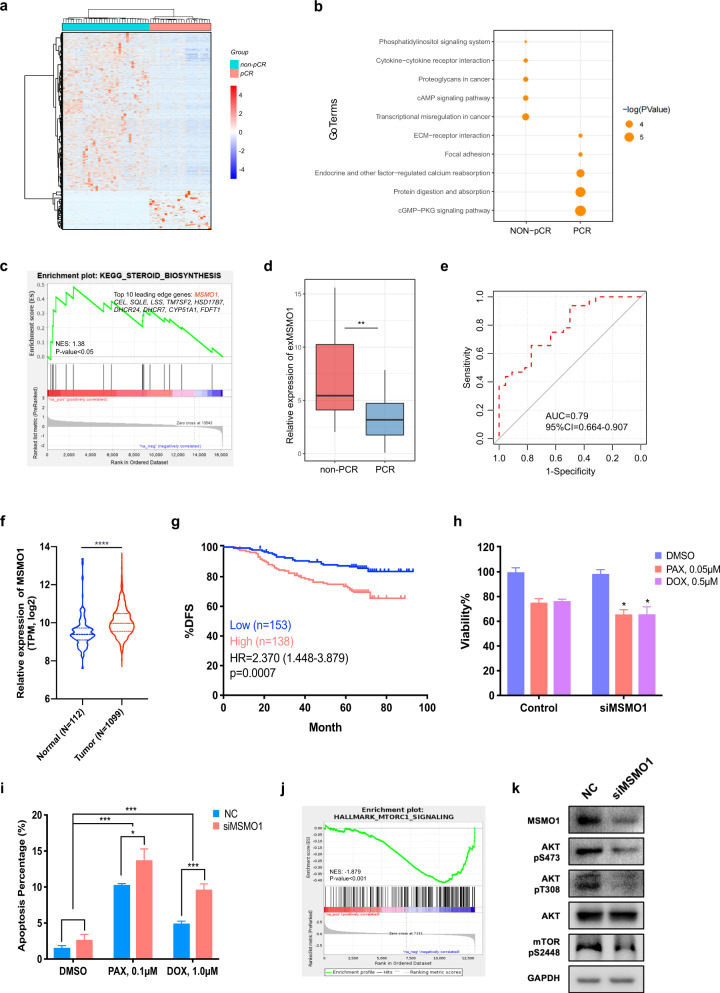
Plasma exMSMO1 as a predictive biomarker for neoadjuvant chemotherapy of BC. **a** Heatmap of different exLRs expressions between pCR (*n* = 24) and non-pCR (*n* = 34) groups. **b** KEGG pathway enrichment analysis for the differentially expressed exLRs of (A). **c** The steroid biosynthesis pathway was enriched in the non-pCR group by gene set enrichment analysis (GSEA). **d** Comparison of exMSMO1 between pCR and non-pCR groups. **e** ROC for the performance of exMSMO1 in predictive neoadjuvant chemotherapy treatment efficacy of BC. **f** Higher expression of MSMO1 in BC tumor tissue compared to adjacent normal in the TCGA database. **g** Kaplan–Meier survival analysis (log-rank test) of disease free survival of BC patients with low (*n* = 157) or high (*n* = 138) MSMO1 expression. **h** MDA-MB-231 cells made deficient in MSMO1 by the siRNAs pool (siMSMO1-1, siMSMO1-2) were treated with the indicated chemotherapy drugs. Viability data from 3 independent experiments were normalized to control–transfected cells. **i** MDA-MB-231 cells were treated with MSMO1 siRNAs pool followed with PAX or DOX for 24 h, and apoptosis was analyzed with the flow cytometry assay. **h**, **i** Only significant differences were shown. Each column represents averaged results. Bars, SDs. **j** Enrichment plots of the hallmark mTORC1 signaling pathway in MSMO1 deficient cells compared to controls, as identified by GSEA. **k** MDA-MB-231 cells were transfected with the MSMO1 siRNAs pool. Cell lysates were immunoblotted as shown. Abbreviations: ROC receiver operating characteristic, AUC area under the curve, SD standard deviation, CI confidence interval, DMSO dimethylsulfoxide, PAX paclitaxel, DOX doxorubicin. *p < 0.05; **p < 0.01; ***p < 0.001; ****p < 0.0001.

We next investigated the ability of exLR profiling to predict treatment responses in BC patients who received NACT. We enrolled 58 local advanced BC (LABC) patients received NACT (paclitaxel (PAX) and/or doxorubicin (DOX) -base regimens, plus trastuzumab if HER2 positive), and 24 achieved a pathological complete response (pCR) after NACT. We identified 2573 exLRs that were differentially expressed in the pCR group compared with the non-pCR group by the Mann–Whitney U test (*p* < 0.05, |FC | > 2). Different expressed exLRs are shown in Fig. 6a. DAVID GO analysis revealed that the increased exLRs were enriched for biological processes such as transcriptional misregulation and proteoglycans in cancer, while decreased exLRs were strongly involved in focal adhesion and cGMP−PKG signaling pathway in non-pCR group (Fig. 6b). Gene set enrichment analysis (GSEA) analyses revealed that the steroid biosynthesis pathway was one of the most upregulated biological processes in the non-pCR group (Fig. 6c).

Methylsterol monooxygenase 1 (MSMO1), an intermediate enzyme in the cholesterol biosynthetic pathway, was identified based on its relative high enrichment in the non-pCR group (*p* < 0.001; Fig. 6d). Extracellular vehicle MSMO1 mRNA (exMSMO1) distinguished non-pCR from pCR patients with an AUC of 0.79 (95% CI: 0.664 to 0.907, Fig. 6e). MSMO1 was highly expressed in tumor tissue of BC compared with adjacent normal tissue in the cancer genome atlas (TCGA) dataset (Fig. 6f). We also evaluated the expression of MSMO1 by the qRT-PCR assay in an independent BC cohort from Fudan University Shanghai Cancer Center (FUSCC, Supplementary Table 2), and we found that high MSMO1 expression was significantly associated with poor disease-free survival (DFS, *p* = 0.0007, Fig. 6g). Multivariate analysis also demonstrated that high MSMO1 expression was independent of unfavorable prognostic factors for DFS in BC patients (hazard ratio (HR) = 2.683; 95% CI: 1.571–4.583; *p* < 0.001, Supplementary Table 3). We performed a functional study using a small interference RNAs (siRNAs) pool and found that the silencing of MSMO1 could significantly enhance the sensitivity of MDA-MB-231 cells to the chemotherapy drug PAX (0.05 μM) and DOX (0.5 μM) (Fig. 6h). Moreover, the inhibition of MSMO1 expression increased the apoptosis rates of MDA-MB-231 to PAX (0.1 μM) and DOX (1.0 μM) (Fig. 6i).

To better understand how MSMO1 promotes drug resistance in breast cancer cells, RNA-seq was performed to analyze the gene expression profile affected by MSMO1 knockdown. GSEA showed that MSMO1 knockdown affected multiple signaling pathways, such as the mTORC1 signaling pathway (Fig. 6j). Further analysis showed that silencing MSMO1 reduced the phosphorylation of AKT and mTOR (Fig. 6k). Considering that mTORC1 signaling activation has been shown to promote therapy resistance in BC^19,20^, these data suggested that MSMO1 could promote drug resistance through modulating the mTORC1 signaling pathways in BC cells.

## Discussion

Thus far, a clinically actionable biomarker that serves diagnosis and monitoring is not available for early BC. In our study, we report that molecular interrogation of blood exosomal long RNA can offer valuable diagnostics information for BC patients. We obtained exLR-seq expression profiles from 172 human plasma EV samples. Differences in the exLR levels were then compared between patients with BC, patients with breast benign disease and healthy participants, and a diagnostic signature for BC was finally established. In addition, the exLRs, such as exMSMO1, can be employable as predictive biomarkers upon response to NACT for LABC.

The latest blood-based biomarkers were evaluated by many scientists to develop novel diagnosis methods. CTCs are rare in localized BC and difficult to isolate, so cementing a role for them in diagnosis appears challenging. Recently, the healthcare biotechnology company GRAIL Inc produce a multicancer early detection assay that identifies abnormally methylated ctDNA with high specificity^21^. The detail data on breast cancer specifically have not been published yet. Moreover, some studies have suggested that tumor-derived EVs represent an appealing source of diagnostic biomarkers^22,23^. The analysis of EVs encompasses several advantages over CTCs and ctDNA due to their higher abundancies and stability in the bloodstream, as well as their functionality in supporting tumor-host cross talk or tumorigenesis.

Until Valadi et al. demonstrated that variable RNAs can be transported between cells by EVs in 2007, EVs have begun to attract the attention of scientists^24^. Recent studies have suggested that some exLRs were differentially expressed between cancer and healthy controls and could have potential for cancer diagnosis^25,26^. To determine the differences in the plasma exLR profiles among BC, breast benign disease and healthy, we performed exLR profiling of plasma samples from all 172 participants using an optimized exLR-seq strategy we recently developed^17^. We identified 1552 exLRs that were differentially expressed in the BC samples compared with controls (benign + healthy) by the Mann–Whitney U test. Then, we integrated the random forest algorithm and the LASSO method and selected eleven exLR markers to construct a BC classifier. The d-signature can distinguish BC from controls with high diagnostic accuracy in both training and validation cohorts (92.5% and 92.3%, respectively). Moreover, it can act as an effective classifier to identify early stage (I/II) BC from controls (AUC = 0.94). The diagnosis of early stage BC is critical in clinical practice, which could enable immediate surgery, thereby improving the prognosis of BC.

Considering the selection result of predicting signature may strongly depend on the arbitrary selection of variable filtering methods, we repeated the data analysis procedures of marker screening and diagnostic model construction without the candidate diagnostic targets enrollment. We firstly filtered 11 exLRs (PPIAP11, DEFA3, RF00002, AL138900.3, MS4A3, chr1_15717893_15721388_+, chr14_86028369_86041833_+, chr17_48112031_48113401_+, chr17_36952886_36953907_+, chr18_12999421_13030608_+, chr6_4891713_4892379_+) to establish a new classifier with concordant parameter tuning processes. Comparing with previously included 11 signature genes, the model based on these new targets showed insufficient efficiency for cancer screening (AUC; training cohort: 0.43 vs. 0.96; validation cohort: 0.94 vs. 0.9). Additionally, we randomly selected the varying number of exLR targets (e.g. 10, 20, 50, 100) to constructed the diagnostic model in the same manner. As a result, the model based on these new targets showed insufficient efficiency for cancer diagnosis in the validation cohort (exLR NO. = 10, 20, 50, 100; AUC = 0.59, 0.47, 0.59, 0.54), which do not lead to a strength classification power. These results indicated that although the cut-off number of candidate targets selection were partially artificial, the predict strength of BRCA classifier based on candidate targets was consistently used to explore the causal connection between selected variables and patient’s outcome.

Clinical doctors routinely choose invasive methods, either core needle biopsy or excision biopsy, to further examine patients who have a BI-RADS 4a or higher finding based on mammography or ultrasound. Wiratkapun et al.^27^ reported that the biopsy rate categorized as BI-RADS 4 was 75% and the diagnosis accuracy was 20.6% in their study. Jung et al.^28^ retrospectively reviewed consecutive core needle biopsy performed from 2005 to 2016 at their institution and found that more than half (58.5%) patients with BI-RADS 4a received biopsy and only 6.7% were diagnosis with malignancy. Thus, if all patients with BI-RADS 4a findings receive invasive procedures, most of the lesions will be benign, which indicates unnecessary treatment for these patients. When integrating the diagnosis exLRs signature with BI-RADS 4a or higher findings, the diagnosis accuracy was approximately 91.9%. Moreover, if we considered only the patients with BI-RADS 4a or 4b, the integrated predictive value was approximately 91.3%. Therefore, by integrating the exLRs signature and film findings, the possibility of false positive and the unnecessary treatment of patients would be greatly decreased.

The development of novel prognostic biomarkers for cancer treatment monitoring is of great valuable in clinical practice. Some studies suggested that certain functional exLRs were valuable for the prediction of different cancer treatment responses^29–31^. In terms of BC, Koldemir et al. showed that the cellular expression of lncRNA GAS5 in BC cells leads to its exosomal enrichment, which is considered to be a marker of apoptotic induction^32^. Moreover, exosomal lncRNA-SNHG14 promotes trastuzumab resistance in HER2-positive BC^33^. However, little is known about the prognostic roles of exLRs in BC treatment. Therefore, to further explore the role of exLRs in BC therapeutic evaluation, we collected and divided 58 NACT patients into pCR (*n* = 24) and non-pCR (*n* = 34) groups based on the post-surgical pathology. Different exLR profiles were found between these two groups. Through GSEA analysis, the steroid biosynthesis pathway is one of the most upregulated pathways in the non-pCR group. Many studies have implicated the function of the sterol synthesis pathway in tumor growth and response to treatment. For example, the sterol composition of the membrane has been shown to regulate EGFR signaling^34^ and the sensitivity of head and neck cancer cells to apoptosis^35^. ExMSMO1 enriched in the non-pCR group and could distinguish non-pCR from pCR with an AUC of 0.79 (95% CI: 0.62 to 0.97, SD: 0.09) (Fig. 6d, e). MSMO1 is an intermediate enzyme in the cholesterol biosynthetic pathway. Previous study has indicated that the inactivation of MSMO1 markedly sensitized tumor cells to therapeutic anti-EGFR antibody via increased EGF receptor degradation^36^. In this study, we demonstrated that MSMO1 was overexpressed in BC and correlated with poor survival. Moreover, MSMO1 can decrease the sensitivity of BC cells to PAX and DOX by regulating the mTORC1 signaling pathway. In other words, these findings demonstrate that exMSMO1 can act as a predictive biomarker for neoadjuvant treatment efficacy of BC.

Several limitations in this study warrant mention. First, as a single-center study, it is uncertain whether this diagnosis signature is applicable to other populations composed of different cultures and ethnic groups. Future work with larger cohorts from multiple centers is still needed to externally validate our results. Second, the role of exLRs in treatment efficient prediction is still not fully evaluated in this study. Further work must be done on this issue.

Our study evaluated the exLRs profiles among BC, benign, and healthy samples and developed a stable and valuable SVM classifier model to distinguish BC and controls. Moreover, we found that certain exLRs, such as exMSMO1, could serve as non-invasive predictive treatment effect biomarkers for BC. We believe that the development of novel effective, noninvasive diagnostic and treatment efficacy predictive tools could be helpful for clinicians.

## Methods

### Patients and clinical features

One hundred and seventy-two participants, including patients with BC (*n* = 112), benign patients (*n* = 19), and healthy controls receiving routine healthcare (healthy, *n* = 41), were enrolled in this study. In addition, the 24 pCR and 34 non-pCR samples were also included to explore the potential application of candidate exLR for therapeutic evaluation. All of the enrolled patients were suspicious for malignancy based on clinical or radiological evidence, and they were diagnosed with BC or benign by pathological examination. All of the participants were recruited from FUSCC between 1 July 2017 and 30 December 2018. This study is conducted in accordance with Declaration of Helsinki. Informed written consent was obtained from each subject, and the study was approved by Institutional Review Board of FUSCC, China.

### Plasma sample collection

Blood samples were collected with 10 mL EDTA-coated Vacutainer tubes from all participants. Blood samples were collected before surgery from early stage BC patients and before chemotherapy from metastatic or LABC patients. Plasma was separated by centrifugation at 800 × *g* (~3000 rpm) for 10 min at room temperature (25 °C) within 2 h after blood collection. This step was followed by a second 10 min centrifugation at 16,000×*g* (~13 000 rpm) at 4 °C to remove the cellular debris. Plasma samples were aliquoted and stored at –80 °C until use.

### Isolation of EVs and EV RNAs

For each patient, 1 mL of fresh or once-frozen thawed plasma was used, and EVs were isolated by affinity-based binding to spin columns using an exoRNeasy Serum/Plasma Kit (Qiagen, Hilden, Germany) following the manufacturer’s instructions. Briefly, thawed plasma was mixed with binding buffer and added to the exoEasy membrane affinity spin column. For TEM, size distribution measurement and Western blotting, the EVs were eluted with 400 μL of XE elution buffer. To concentrate the EVs, samples were subjected to ultrafiltration using the Amicon Ultra-0.5 Centrifugal Filter 10 kDa (Merck Millipore, Germany). For the EV RNA isolation, EVs were lysed on the column using QIAzol (Qiagen), and the total RNA was then eluted and purified.

### Transmission electron microscopy

EVs were identified by negative staining with phosphotungstic acid. Fifty microliters of resuspended EVs were placed on a Parafilm membrane. A copper mesh with a formvar supporting membrane was covered with the EVs suspension and floated for 3–10 min to allow sample absorption into the supporting membrane. Next, 50 μL of 2% phosphotungstic acid was dropped onto the Parafilm membrane. The fluid was then absorbed from the edges of the copper mesh with filter paper. The copper mesh absorbing the sample was covered with 2% phosphotungstic acid and floated for 3 min. Then, the sample was dried for 10 min under incandescent light after the staining solution was absorbed with filter paper. The copper mesh was imaged with a TEM (Phillips CM120, Tokyo, Japan).

### Size distribution measurement

Size distribution analysis of the EVs was performed with a Flow NanoAnalyzer (NanoFCM Inc., Xiamen, China) according to the manufacturer’s instructions. A series of monodisperse silica nanoparticles (SiNPs) were synthesized and used as size reference standards. Then, the side scattering (SSC) distribution histogram of the mixture was obtained. The SSC intensity of every vesicle was converted into its corresponding vesicle size. One hundred mL phosphate-buffered saline (PBS) resuspended EV samples and 100 mL PBS (blank control) were analyzed using the same instrument settings. The EV data were analyzed and used to construct a size distribution histogram.

### Western blotting of EVs

PBMCs were isolated by Lymphoprep (STEMCELL Technologies, USA) according to the manufacturer’s instructions. PBMCs and the concentrated EVs were lysed in RIPA buffer (1% NP40, 0.5% deoxycholate, 0.1% sodium dodecyl sulfate [SDS] in Tris-buffered saline) with complete protease inhibitors on ice for 30 min. Equal amounts of protein from EVs and PBMC were separated on 10% SDS-polyacrylamide gels and then transferred to nitrocellulose membranes (Bio-Rad, Hercules, CA, USA). Membranes were blocked with 5% non-fat milk and incubated with anti-CD63 (Cat# 216130, Abcam; 1: 1,000) and anti-TSG101 (Cat# 136111, Santa Cruz Biotechnology; 1:500). Target proteins were detected using an enhanced chemiluminescence kit (Amersham Pharmacia Biotech, Uppsala, Sweden).

### Cell lines and compounds

MDA-MB-231 cell line was obtained from the American Type Culture Collection (ATCC) which was characterized by Short Tandem Repeat (STR) profiling. Cells resuscitated from original passage and passaged within 6 months were used in all experiments. All these cells were cultured under standard conditions. PAX and DOX were purchased from Selleck.

### siRNA transfections and in vitro viability assays

The siRNAs targeting MSMO1 and controls were obtained from RioBio. Sequences of effective siRNA are as follows:

siMSMO1-1: 5’- GAACAGACUCUCAGUAUAAdTdT-3’;

siMSMO1-2: 5’- GCUGUGGAAUAUGUAGAUUdTdT-3’.

Cells were transfected in triplicates with siRNAs pool at 10 nmol/L concentrations mixed with Lipofectamine RNAiMAX reagent (Thermo Scientific) on a 96-well plate according to the manufacturer’s reverse transfection protocol. Twenty-four hours after plating, cells were treated with PTX (0.05 μM), DOX (0.5 μM), or dimethylsulfoxide (DMSO, 0.02%). The viability was measured in 48 h using CCK-8 (Promega).

### Apoptosis and pathway analysis

Apoptosis was measured using the Annexin V assay (Guava Technologies). Annexin V–positive MDA-MB-231 cells were counted using flow cytometry 72 h after transfection, 48 h after PTX (0.1 μM), DOX (1.0 μM), or vehicle treatment. Representative flow cytometry sequential gating/sorting strategies were shown in Supplementary Fig. 1. Cell extracts were prepared using Tissue Protein Extraction buffer (T-PER) (Thermo Scientific) supplemented with the Halt Phosphatase Inhibitor Cocktail and the Complete Mini Protease Inhibitor Cocktail (Thermo Scientific). Extracts were centrifuged at 15,000 × *g* for 10 min at 4 °C. Western blot analysis was conducted using antibodies to phosphorylated and total AKT and mTOR and to GAPDH. The following primary antibodies were commercially obtained: MSMO1 (Cat# 46773, Sigma-Aldrich; 1:1,000), Phospho-Akt (Ser473) (Cat# 9271, Cell Signaling Technology; 1:1,000), Phospho-Akt (Thr308) (Cat# 9275, Cell Signaling Technology; 1:1,000), Akt (Cat# 9272, Cell Signaling Technology; 1:1,000), Phospho-mTOR (Ser2448) (Cat# 2971, Cell Signaling Technology; 1:1,000), and GAPDH (Cat# 125247, Abcam; 1:1,000). All blots derive from the same experiment and were processed in parallel.

### RNA-seq analysis

Total EV RNA isolated from 1 mL of plasma was treated with DNase I (NEB, Ipswich, Massachusetts, USA) to remove DNA. Strand-specific RNA-seq libraries were prepared using the SMARTer Stranded Total RNA-Seq Kit—Pico Input Mammalian (Clontech, Palo Alto, California, USA). The library quality was analyzed using a Qubit fluorometer (Thermo Scientific, Waltham, Massachusetts, USA) and Qsep100 (BiOptic, New Taipei City, Taiwan). EV RNA-seq libraries could be prepared from 1 mL of plasma on average. ExLR-seq was performed on an Illumina sequencing platform (San Diego, California, USA) with 150 bp paired-end run metrics.

Raw reads were filtered using FastQC and aligned to the GRCh38 human genome assembly using STAR. Annotations of mRNA and lncRNA in the human genome were retrieved from the GENCODE (V.25). The circRNAs were discovered by the Assembling Splice Junctions Analysis (ASJA)^37^, and the normalized method was the same as in a previous study^17^. The length and backsplicing ratio of the circRNAs were calculated on the basis of previous study^37^. Gene expression levels were calculated in transcripts per kilobase million (TPM). Differentially regulated exLRs were annotated gene IDs and were assessed for KEGG pathway enrichment using DAVID (https://david.ncifcrf.gov/).

### Data and statistical analyses

RNA-seq raw read counts were converted to TPM values to scale all of the comparable variates and were normalized across all samples. Variates with frequencies of <10% (i.e., expressed in less than 10% of the entire samples) were omitted, and the remaining markers were used for subsequent statistical analyses.

We used the Random Forest to find markers that could distinguish BC and control (benign + healthy) samples. The steps were as followed: (1) ExLR-seq TPM expression profiles (*n* = 172) were randomly distributed in training (*n* = 120) and validation cohorts (*n* = 52). (2) In the training cohort, the Mann–Whitney U test was used to assess the differential expression of exLRs in BC and control cohorts, and the *p* value of each marker was adjusted by the Benjamini–Hochberg method to control the FDR. (3) The RandomForestClassifier method of the sklearn package and 0.01 threshold of SelectFromModel were used to define candidate makers and accuracy score. (4) Repeat (1) to (3) steps for 3000 times. Finally, we selected the best candidate makers based on accuracy score and then calculated the out-of-bag (OOB) error to determine markers. We received the learning curve of AUC from the classifiers after thousands time of fitting, and the desired plateau condition in curve was observed (Mean = 0.85, SD = 0.03), indicates the model has strong adaptability to the new data set, and the sample size is sufficient.

Eleven exLRs evaluated by the mentioned algorithms and annotations were selected to construct a SVM model for BC prediction. For binary (BC vs benign + healthy) sample classification, the SVM algorithm was executed using the ‘LinearSVC’ package in python software. In principle, the SVM algorithm determines the location of all samples in a high-dimensional space, in which each axis represents an exLR and the expression level of particular exLR in a sample determines its location on the axis. We divided sample randomly into training (*n* = 120) and validation (*n* = 52) cohorts with ratio of 7:3. During the training process, the SVM algorithm draws a hyperplane that best separates the two classes based on the distance between the closest sample of each class and the hyperplane. The different sample classes are positioned at each side of the hyperplane. Moreover, to assess the predictive value of the SVM algorithm in an independent data set, which is not typically included in the SVM training process, the algorithm was trained with the training data set, all SVM parameters were fixed and samples in the internal validation cohorts were then evaluated. The internal training performance of the SVM algorithm could be improved by enabling the SVM tuning function, which implies optimal determination of parameters of the SVM algorithm (C, gamma, kernel) by randomly subsampling the data set used for the algorithm training (‘fivefold internal cross-validation’).

The d-signature score was computed from the predictive strength of the SVM classifier output. To assess the samples’ probability of being predicted as BC, we used the R function ‘predict’ to evaluate the prediction strength in quantitative terms on the internal validation. The prediction strength of the SVM classifier output was used to establish the exLR d-signature. The diagnostic efficacy of the d-signature was evaluated by receiver operating characteristic (ROC) curve analysis for the training, internal validation. The comparison between the AUCs of the different classifiers was evaluated by the bootstrap method with 3000 iterations. Youden’s index was determined to identify the optimal cut-off point for calculating the exact diagnostic indices. The d-signature distribution in the different patient groups was tested by the Wilcoxon rank-sum test and Student’s *t* test after using the Shapiro–Wilk test to determine the data normality.

For most of the experiments, independent sample *t*-tests were used to calculate the *p* values. DFS was derived from the Kaplan–Meier estimate and compared by the log-rank test. Cox regression analysis was performed to assess the effect of potential risk factors upon the survival time.

All statistical analyses were two-sided, and a *p* value <0.05 was considered to be statistically significant. R software packages (‘varSelRF’ and ‘pROC’) and python packages (‘RandomForestClassifier’, ‘train_test_split’, ‘SelectFromModel’, ‘accuracy_score’, ‘StratifiedKFold’ and ‘LinearSVC’) were used in this study.

### Reporting summary

Further information on research design is available in the Nature Research Reporting Summary linked to this article.

## Supplementary information

Supplementary Information

Reporting Summary

## Data Availability

RNA-seq datasets were uploaded to Genome Sequence Archive for Human (GSA-Human) under accession number HRA001985 and can be accessed using the following link: https://bigd.big.ac.cn/gsa-human/browse/HRA001985. Additional datasets used and/or analyzed during the current study are available from the corresponding author on reasonable request.

## Code availability

Detailed algorithm and software for data analysis can be found in the methods section. The code developed during the current study are available upon reasonable request.

## References

[1] Siegel RL, Miller KD, Jemal A (2019). Cancer statistics, 2019. CA Cancer J. Clin..

[2] DeSantis CE (2019). Breast cancer statistics, 2019. CA Cancer J. Clin..

[3] Chan CH, Coopey SB, Freer PE, Hughes KS (2015). False-negative rate of combined mammography and ultrasound for women with palpable breast masses. Breast Cancer Res. Treat..

[4] Harding C (2015). Breast cancer screening, incidence, and mortality across US counties. JAMA Intern. Med..

[5] Narod S (2016). Breast cancer: the importance of overdiagnosis in breast-cancer screening. Nat. Rev. Clin. Oncol..

[6] Ohuchi N (2016). Sensitivity and specificity of mammography and adjunctive ultrasonography to screen for breast cancer in the Japan Strategic Anti-cancer Randomized Trial (J-START): a randomised controlled trial. Lancet.

[7] Ya-jie J (2013). Application of breast ultrasound in a mammography-based Chinese breast screening study. Cell Biochem. Biophys..

[8] Mirabelli P, Incoronato M (2013). Usefulness of traditional serum biomarkers for management of breast cancer patients. BioMed Res. Int.

[9] Radovich M (2020). Association of circulating tumor DNA and circulating tumor cells after neoadjuvant chemotherapy with disease recurrence in patients with triple-negative breast cancer: preplanned secondary analysis of the BRE12-158 randomized clinical trial. JAMA Oncol..

[10] Cavallone L (2020). Prognostic and predictive value of circulating tumor DNA during neoadjuvant chemotherapy for triple negative breast cancer. Sci. Rep..

[11] Magbanua MJM (2021). Circulating tumor DNA in neoadjuvant-treated breast cancer reflects response and survival. Ann. Oncol..

[12] McDonald, B. R. et al. Personalized circulating tumor DNA analysis to detect residual disease after neoadjuvant therapy in breast cancer. *Sci. Transl. Med*. **11**, eaax7392 (2019). 10.1126/scitranslmed.aax7392 PMC7236617 31391323

[13] Bidard FC (2018). Circulating tumor cells in breast cancer patients treated by neoadjuvant chemotherapy: a meta-analysis. J. Natl Cancer Inst..

[14] Colombo M, Raposo G, Thery C (2014). Biogenesis, secretion, and intercellular interactions of exosomes and other extracellular vesicles. Annu Rev. Cell Dev. Biol..

[15] van Niel G, D’Angelo G, Raposo G (2018). Shedding light on the cell biology of extracellular vesicles. Nat. Rev. Mol. Cell Biol..

[16] Rodriguez-Martinez A (2019). Exosomal miRNA profile as complementary tool in the diagnostic and prediction of treatment response in localized breast cancer under neoadjuvant chemotherapy. Breast Cancer Res..

[17] Li Y (2019). Extracellular vesicles long RNA sequencing reveals abundant mRNA, circRNA, and lncRNA in human blood as potential biomarkers for cancer diagnosis. Clin. Chem..

[18] Li Y (2015). Circular RNA is enriched and stable in exosomes: a promising biomarker for cancer diagnosis. Cell Res..

[19] Jiang BH, Liu LZ (2008). Role of mTOR in anticancer drug resistance: perspectives for improved drug treatment. Drug Resist. Updat..

[20] Butt G (2019). Role of mTORC1 and mTORC2 in breast cancer: therapeutic targeting of mTOR and its partners to overcome metastasis and drug resistance. Adv. Exp. Med. Biol..

[21] Oxnard GR, Klein EA, Seiden M, Hubbell E, Liu MCJJOGO (2019). Simultaneous multi-cancer detection and tissue of origin (TOO) localization using targeted bisulfite sequencing of plasma cell-free. DNA.

[22] Melo SA (2015). Glypican-1 identifies cancer exosomes and detects early pancreatic cancer. Nature.

[23] Yang, K. S. et al. Multiparametric plasma EV profiling facilitates diagnosis of pancreatic malignancy. *Sci. Transl. Med*. **9**, eaal3226 (2017). 10.1126/scitranslmed.aal3226 PMC5846089 28539469

[24] Valadi H (2007). Exosome-mediated transfer of mRNAs and microRNAs is a novel mechanism of genetic exchange between cells. Nat. Cell Biol..

[25] Goldvaser H (2017). Characterisation of blood-derived exosomal hTERT mRNA secretion in cancer patients: a potential pan-cancer marker. Br. J. Cancer.

[26] Yu S (2020). Plasma extracellular vesicle long RNA profiling identifies a diagnostic signature for the detection of pancreatic ductal adenocarcinoma. Gut.

[27] Wiratkapun C, Bunyapaiboonsri W, Wibulpolprasert B, Lertsithichai P (2010). Biopsy rate and positive predictive value for breast cancer in BI-RADS category 4 breast lesions. J. Med. Assoc. Thai.

[28] Jung I (2020). Annual trends in ultrasonography-guided 14-gauge core needle biopsy for breast lesions. Korean J. Radio..

[29] Del Re M (2017). The detection of androgen receptor splice variant 7 in plasma-derived exosomal RNA strongly predicts resistance to hormonal therapy in metastatic prostate cancer patients. Eur. Urol..

[30] Del Re M (2018). PD-L1 mRNA expression in plasma-derived exosomes is associated with response to anti-PD-1 antibodies in melanoma and NSCLC. Br. J. Cancer.

[31] Qu L (2016). Exosome-transmitted lncARSR promotes sunitinib resistance in renal cancer by acting as a competing endogenous RNA. Cancer Cell.

[32] Koldemir O, Ozgur E, Gezer U (2017). Accumulation of GAS5 in exosomes is a marker of apoptosis induction. Biomed. Rep..

[33] Dong H (2018). Exosome-mediated transfer of lncRNASNHG14 promotes trastuzumab chemoresistance in breast cancer. Int J. Oncol..

[34] Sigismund S (2008). Clathrin-mediated internalization is essential for sustained EGFR signaling but dispensable for degradation. Dev. Cell.

[35] Bionda C (2008). Differential regulation of cell death in head and neck cell carcinoma through alteration of cholesterol levels in lipid rafts microdomains. Biochem. Pharm..

[36] Sukhanova A (2013). Targeting C4-demethylating genes in the cholesterol pathway sensitizes cancer cells to EGF receptor inhibitors via increased EGF receptor degradation. Cancer Discov..

[37] Zhao J (2019). ASJA: a program for assembling splice junctions analysis. Comput. Struct. Biotechnol. J..

