# Author Correction: Plasma extracellular vesicle long RNA profiles in the diagnosis and prediction of treatment response for breast cancer

**DOI:** 10.1038/s41523-022-00408-y

**Published:** 2022-03-15

**Authors:** Yonghui Su, Yuchen Li, Rong Guo, Jingjing Zhao, Weiru Chi, Hongyan Lai, Jia Wang, Zhen Wang, Lun Li, Yuting Sang, Jianjing Hou, Jingyan Xue, Zhimin Shao, Yayun Chi, Shenglin Huang, Jiong Wu

**Affiliations:** 1 Department of Breast Surgery, Fudan University Shanghai Cancer Center, and the Shanghai Key Laboratory of Medical Epigenetics, Institutes of Biomedical Sciences, Fudan University, Shanghai, 200032 P. R. China; 2 Shanghai Key Laboratory of Breast Cancer, Fudan University Shanghai Cancer Center, Fudan University, Shanghai, 200032 P. R. China; 3 Department of Oncology, Shanghai Medical college, Fudan University, Shanghai, 200032 P. R. China; 4 Shanghai Key Laboratory of Radiation Oncology, Fudan University Shanghai Cancer Center, Shanghai, 200032 P. R. China

**Keywords:** Diagnostic markers, Breast cancer

Correction to: *npj Breast Cancer* 10.1038/s41523-021-00356-z, published online 10 December 2021

The original version of the published Article had an error in the Data availability statement. The Genome Sequence Archive for Human accession number for the RNA-seq data has been corrected to HRA001985. The HTML and PDF versions of the Article have been corrected.

