# Supplementary material for: Plasma extracellular vesicle long RNA profiles in the diagnosis and prediction of treatment response for breast cancer
Source: NPJ Breast Cancer. 2021 Dec 10;7:154. doi: 10.1038/s41523-021-00356-z (PMC8664804; doi:10.1038/s41523-021-00356-z)
Supplement: Supplementary file 1 — Supplementary Information [file 41523_2021_356_MOESM1_ESM.pdf]

Supplementary Table 1. Characteristics of the markers for BC detection identified in training set.

Gene	Type	P (wilcox test)	Fold change (mean)	Fold change (median)
BEX2*	Protein coding	0.000310028	1.551658348	1.630823933
AC104843.1	Processed pseudogene	1.46E-05	1.9550703	2.181653303
AL136981.2	Unprocessed pseudogene	0.000206439	1.606567296	3.475874025
KRT19*	Protein coding	6.51E-05	2.549444982	11.3287037
NPM1P25*	Processed pseudogene	0.000357388	1.216086475	4.044264819
CTSG*	Protein coding	0.000410313	1.872959802	5.614639397
CBR3*	Protein coding	8.25E-05	1.651809737	2.903473492
HOXB7*	Protein coding	0.000166308	1.524901639	1.636319099
AL691447.3	Processed pseudogene	1.82E-05	1.78739331	2.080022052
RNA5SP141	rRNA pseudogene	5.56E-05	1.892021691	Infinity
chr13_42953948_42970670_-	circRNA	0.000460577	1.688474834	2.761911793

* Differentially expressed genes in the Cancer Genome Atlas Breast Cancer

Supplementary Table 2. Relationship between MSMO1 expression and clinicopathological features in 291 primary BC patients.

Characteristics	Low (%) n=153	High (%) n=138	*p*-value
Age(years)			0.104
≤ 45	36 (23.5)	35 (25.4)	
> 45 and ≤ 60	86 (56.2)	62 (44.9)	
> 60	31 (20.3)	41 (29.7)	
Menopausal status*			0.766
Pre	63 (41.2)	52 (37.7)	
Post	87 (56.9)	84 (60.9)	
Grade*			0.763
G1/2	90 (58.8)	81 (58.7)	
G3	51 (33.3)	49 (35.5)	
pT stage*			0.692
T1	56 (36.6)	46 (33.3)	
T2/3	96 (62.7)	90 (65.2)	
pN stage			0.854
N0	64 (41.8)	61 (44.2)	
N1	50 (32.7)	39 (28.3)	
N2	16 (10.5)	17 (12.3)	
N3	23 (15.0)	21 (15.2)	
LVI*			0.307
Negative	85 (55.6)	65 (47.1)	
Positive	65 (42.5)	71 (51.4)	
ER status*			0.590
Negative	45 (29.4)	38 (27.5)	
Positive	107 (69.9)	100 (72.5)	
PR status*			0.403
Negative	34 (22.2)	25 (18.1)	
Positive	119 (77.8)	112 (81.2)	
HER2 status*			0.033
Negative	113 (73.9)	82 (59.4)	
Positive	34 (22.2)	48 (34.8)	
Ki67*			0.272
Low	58 (37.9)	45 (32.6)	
High	35 (22.9)	43 (31.2)	

Abbreviations: pT, pathological tumor size; pN, pathological lymph-node status; LVI, lymphovascular invasion; ER, estrogen receptor; PR, progesterone receptor; HER2, human epidermal growth factor receptor 2;
*Excluded the unknown category.

Supplementary Table 3. Univariate and multivariate Cox regression analyses of DFS in BC patients.

	Univariate analysis		Multivariate analysis	
Covariates	HR (95% CI)	*p*-value	HR (95% CI)	*p*-value
Age (years)		0.425		
41-60 versus ≤40	0.648 (0.371-1.133)	0.128	——	
>60 versus ≤40	0.596 (0.298-1.190)	0.143	——	
Grade (G3 versus G1/2)	1.404 (0.837-2.355)	0.199	——	
pT stage (T2/3 versus T1)	2.113 (1.166-3.828)	0.014	1.668 (0.903-3.081)	0.102
pN stage		<0.001		<0.001
N1 versus N0	2.477 (1.267-4.843)	0.008	3.342 (1.570-7.113)	0.002
N2 versus N0	2.861 (1.237-6.614)	0.014	3.952 (1.553-10.057)	0.004
N3 versus N0	5.276 (2.640-10.544)	<0.001	7.757 (3.192-18.851)	<0.001
LVI (positive versus negative)	2.011 (1.198-3.375)	0.008	0.668 (0.345-1.291)	0.230
ER (positive versus negative)	0.900 (0.526-1.539)	0.700	——	
PR (positive versus negative)	0.769 (0.431-1.374)	0.375	——	
HER2 (positive versus negative)	1.327 (0.761-2.314)	0.319	——	
Ki67 (high versus low)	1.565 (0.854-2.869)	0.147	——	
MSMO1 expression level (high versus low)	2.373 (1.416-3.976)	0.001	2.683 (1.571-4.583)	<0.001

Abbreviations: DFS, disease-free survival; pT, pathological tumor size; pN, pathological lymph-node status; LVI, lymphovascular invasion; ER, estrogen receptor; PR, progesterone receptor; HER2, human epidermal growth factor receptor 2; HR, hazard ratio; CI, confidence interval.

Supplementary Figure 1.

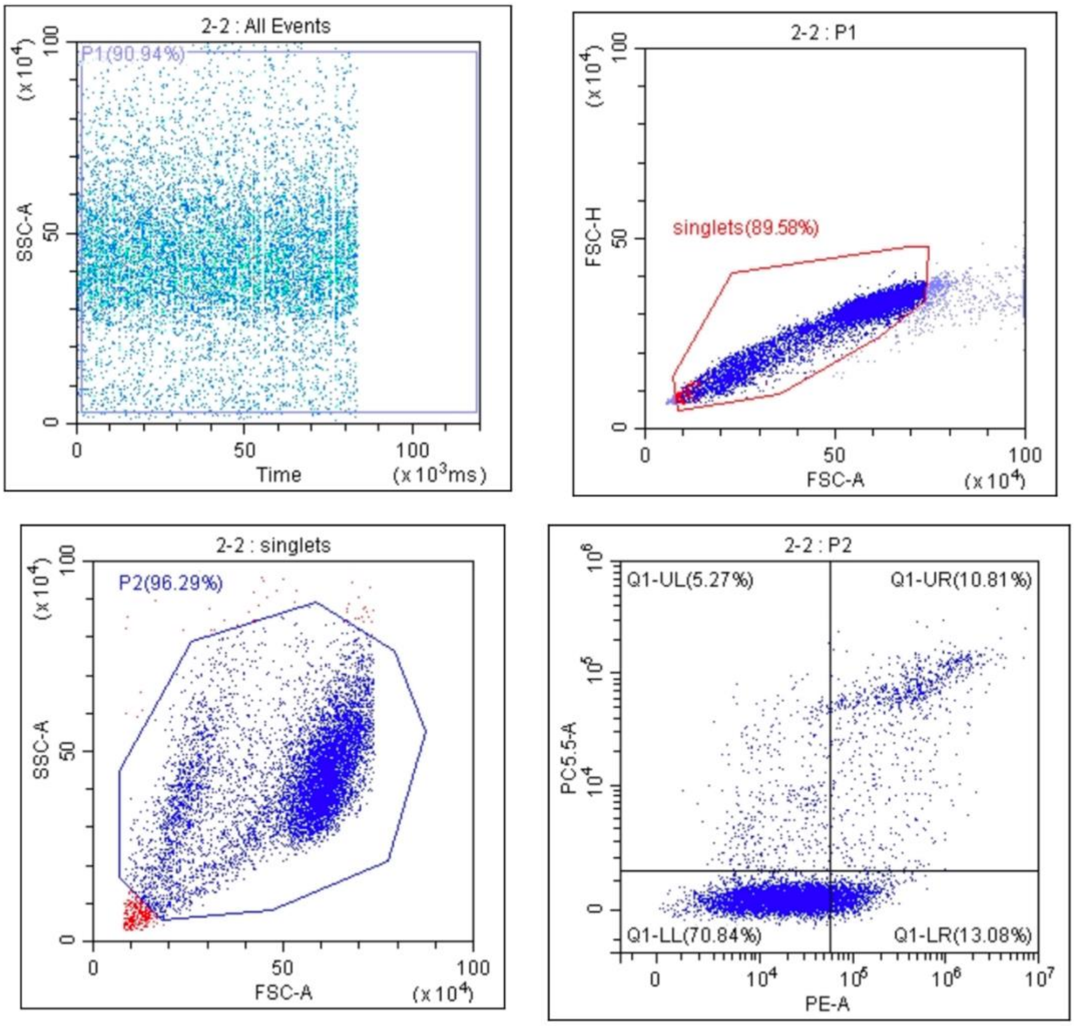

Supplementary Figure 1. Flow cytometry gating strategy example. Single cells were gated based on forward and side scatter properties after the exclusion of dead cells and doublets. Cell viability was then gated according to Annexin V (PE) and 7-AAD (PerCP5.5). The Q1-LR (low right) quadrant indicates the early apoptotic cells which were used in the final statistical analysis (Figure 6i).

SSC-A, side scatter area; FSC-A, forward scatter area; FSC-H, forward scatter height; UL, up low; UR, up right; LL, low left.

Supplementary Figure 2.

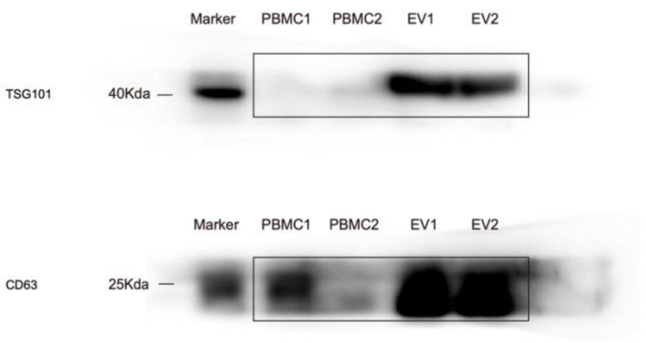

Supplementary Figure 2. Uncropped images of western blots shown in Figure 1c. The protein standards are depicted to the left of the images.

Supplementary Figure 3.

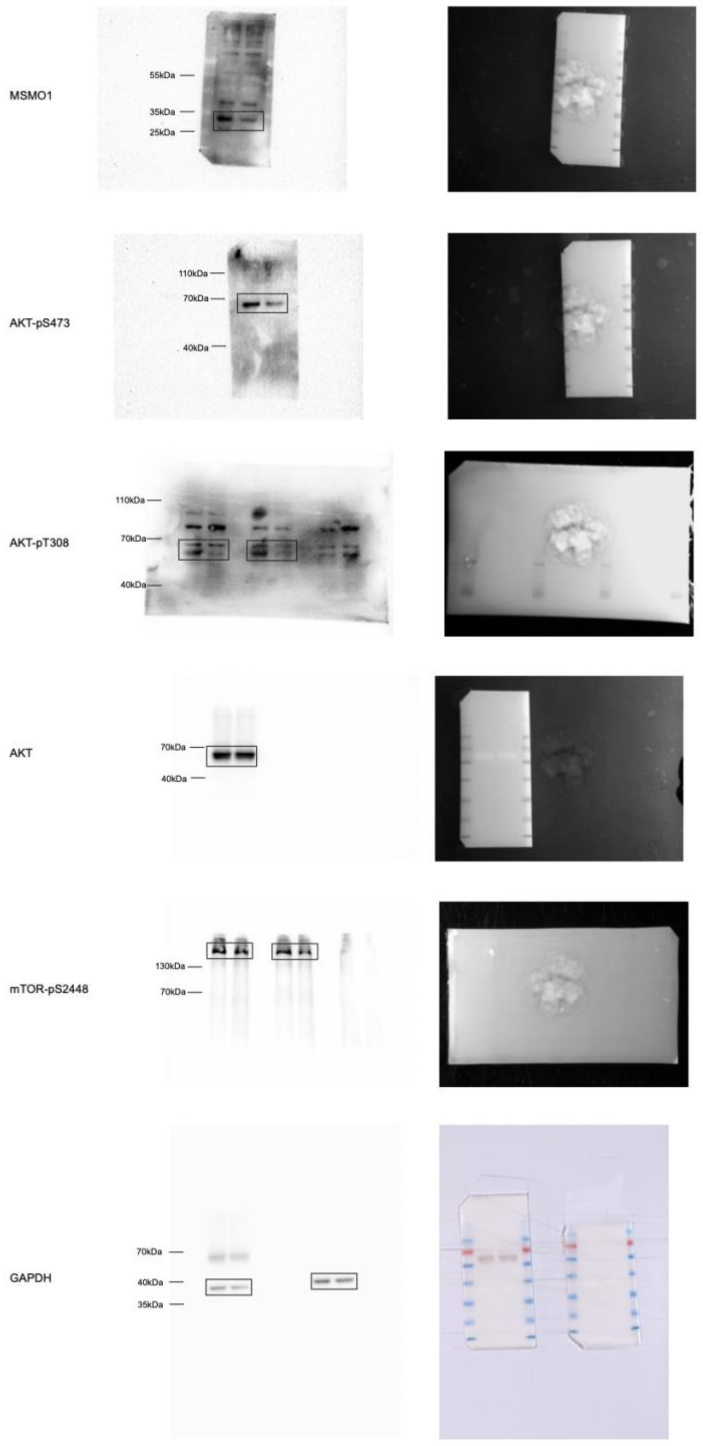

Supplementary Figure 3. Uncropped images of western blots shown in Figure 6k. The protein standards are depicted to the left of the images.
