# Supplementary material for: Plasma extracellular vesicle long RNA profiles in the diagnosis and prediction of treatment response for breast cancer
Source: NPJ Breast Cancer. 2021 Dec 10;7:154. doi: 10.1038/s41523-021-00356-z (PMC8664804; doi:10.1038/s41523-021-00356-z)
Supplement: Supplementary file 2 — Reporting Summary [file 41523_2021_356_MOESM2_ESM.pdf]

nature portfolio

Corresponding author(s): Jiong Wu

Last updated by author(s): Oct 11, 2021

Reporting Summary

Nature Portfolio wishes to improve the reproducibility of the work that we publish. This form provides structure for consistency and transparency in reporting. For further information on Nature Portfolio policies, see our Editorial Policies and the Editorial Policy Checklist.

Statistics

For all statistical analyses, confirm that the following items are present in the figure legend, table legend, main text, or Methods section.

n/a	Confirmed	
☐	☒	The exact sample size (n) for each experimental group/condition, given as a discrete number and unit of measurement
☐	☒	A statement on whether measurements were taken from distinct samples or whether the same sample was measured repeatedly
☐	☒	The statistical test(s) used AND whether they are one- or two-sided<br>*Only common tests should be described solely by name; describe more complex techniques in the Methods section.*
☐	☒	A description of all covariates tested
☐	☒	A description of any assumptions or corrections, such as tests of normality and adjustment for multiple comparisons
☐	☒	A full description of the statistical parameters including central tendency (e.g. means) or other basic estimates (e.g. regression coefficient) AND variation (e.g. standard deviation) or associated estimates of uncertainty (e.g. confidence intervals)
☐	☒	For null hypothesis testing, the test statistic (e.g. F, t, r) with confidence intervals, effect sizes, degrees of freedom and P value noted<br>*Give P values as exact values whenever suitable.*
☒	☐	For Bayesian analysis, information on the choice of priors and Markov chain Monte Carlo settings
☒	☐	For hierarchical and complex designs, identification of the appropriate level for tests and full reporting of outcomes
☒	☐	Estimates of effect sizes (e.g. Cohen's d, Pearson's r), indicating how they were calculated

Our web collection on statistics for biologists contains articles on many of the points above.

Software and code

Policy information about availability of computer code

Data collection	NA
Data analysis	Detailed algorithm and software for data analysis can be found in the methods section.

For manuscripts utilizing custom algorithms or software that are central to the research but not yet described in published literature, software must be made available to editors and reviewers. We strongly encourage code deposition in a community repository (e.g. GitHub). See the Nature Portfolio guidelines for submitting code & software for further information.

Data

Policy information about availability of data

All manuscripts must include a data availability statement. This statement should provide the following information, where applicable:

- Accession codes, unique identifiers, or web links for publicly available datasets
- A description of any restrictions on data availability
- For clinical datasets or third party data, please ensure that the statement adheres to our policy

RNA-seq datasets were uploaded on Genome Sequence Archive (GSA) under the Accession Number of PRJCA004407. Datasets used and/or analyzed during the current study are available from the corresponding author on reasonable request.

Field-specific reporting

Please select the one below that is the best fit for your research. If you are not sure, read the appropriate sections before making your selection.

☒ Life sciences ☐ Behavioural & social sciences ☐ Ecological, evolutionary & environmental sciences

For a reference copy of the document with all sections, see nature.com/documents/nr-reporting-summary-flat.pdf

Life sciences study design

All studies must disclose on these points even when the disclosure is negative.

Sample size	We used the PASS software (version 11) to determinate whether the current sample size harbored adequate power for diagnostic accuracy assay.
Data exclusions	NA
Replication	NA
Randomization	NA
Blinding	NA

Reporting for specific materials, systems and methods

We require information from authors about some types of materials, experimental systems and methods used in many studies. Here, indicate whether each material, system or method listed is relevant to your study. If you are not sure if a list item applies to your research, read the appropriate section before selecting a response.

Materials & experimental systems

n/a	Involved in the study	
☐	☒	Antibodies
☐	☒	Eukaryotic cell lines
☒	☐	Palaeontology and archaeology
☒	☐	Animals and other organisms
☐	☒	Human research participants
☒	☐	Clinical data
☒	☐	Dual use research of concern

Methods

n/a	Involved in the study	
☒	☐	ChIP-seq
☐	☒	Flow cytometry
☒	☐	MRI-based neuroimaging

Antibodies

Antibodies used	CD63 (Cat# 216130, Abcam; 1: 1,000), TSG101 (Cat# 136111, Santa Cruz Biotechnology; 1:500), MSMO1 (Cat# 46773, Sigma-Aldrich; 1:1,000), Phospho-Akt (Ser473) (Cat# 9271, Cell Signaling Technology; 1:1,000), Phospho-Akt (Thr308) (Cat# 9275, Cell Signaling Technology; 1:1,000), Akt (Cat# 9272, Cell Signaling Technology; 1:1,000), Phospho-mTOR (Ser2448) (Cat# 2971, Cell Signaling Technology; 1:1,000), and GAPDH (Cat# 125247, Abcam; 1:1,000).
Validation	NA

Eukaryotic cell lines

Policy information about cell lines

Cell line source(s)	MDA-MB-231 cell line was obtained from ATCC which was characterized by Short Tandem Repeat (STR) profiling.
Authentication	None of cell lines used were authenticated.
Mycoplasma contamination	All cell lines tested negative for mycoplasma contamination.
Commonly misidentified lines (See ICLAC register)	NA

Human research participants

Policy information about [studies involving human research participants]

Population characteristics	All of the enrolled patients were suspicious for malignancy based on clinical or radiological evidence, and they were diagnosed with BC or benign by pathological examination.
Recruitment	All of the participants were recruited from Fudan University Shanghai Cancer Center between 1 July 2017 and 30 December 2018.
Ethics oversight	Informed written consent was obtained from each subject, and the study was approved by Institutional Review Board of Fudan University Shanghai Cancer Center, China.

Note that full information on the approval of the study protocol must also be provided in the manuscript.

Flow Cytometry

Plots

Confirm that:

☒ The axis labels state the marker and fluorochrome used (e.g. CD4-FITC).

☒ The axis scales are clearly visible. Include numbers along axes only for bottom left plot of group (a 'group' is an analysis of identical markers).

☒ All plots are contour plots with outliers or pseudocolor plots.

☒ A numerical value for number of cells or percentage (with statistics) is provided.

Methodology

Sample preparation	Apoptosis was measured using the PE Annexin V apoptosis Detection KIT I (BD PharmingenTM). MDA-MB-231 cells were counted using flow cytometry 72 hours after transfection, 48 hours after PTX, DOX, or vehicle treatment. Briefly, cells were washed twice with cold PBS and then resuspended in 1X Binding buffer at a concentraion of 1X106 cells/ml. Then transfer 100ul of the solution to a 5ml culture tube and add 5ul of PE Annexin V and 5 ul 7-ADD and incubate for 15min at RT in the dark. At last add 400 ul of binding buffer to each tube and analyze by flow cytometry within 1 hr.
Instrument	Cytoflex s instruments (Beckman Coulter)
Software	CytExpert 2.3 software (Beckman Coulter)
Cell population abundance	Not applicable. We didn't do any sorting before analyzing.
Gating strategy	Single cells were gated based on FSC-A/FSC-H, cell viability was then gated according to Annexin V (PE) and 7-AAD (PerCP5.5)

☒ Tick this box to confirm that a figure exemplifying the gating strategy is provided in the Supplementary Information.
